# Obesity and overall mortality: findings from the Jackson Heart Study

**DOI:** 10.1186/s12889-020-10040-9

**Published:** 2021-01-06

**Authors:** Yuan-I Min, Yan Gao, Pramod Anugu, Anshul Anugu, Adolfo Correa

**Affiliations:** The Jackson Heart Study, University of Mississippi Medical Center, 350 W. Woodrow Wilson Avenue, Ste. 701, Jackson, MS 39213 USA

**Keywords:** Obesity, Body mass index, Waist circumference, Waist-to-height ratio, Waist-to-hip ratio, Mortality, Overall mortality, Cardiovascular mortality, Cancer mortality

## Abstract

**Background:**

Overall mortality has been reported to be lower among individuals classified as overweight/obese when compared with their normal weight counterparts (“obesity paradox”) when obesity classification is based on the body mass index (BMI). One possible reason for this apparent paradox is that BMI is not a reliable measure of obesity-related risk as it does not differentiate fat mass from lean muscle mass or fat mass phenotypes. Waist circumference (WC), as a measure of central adiposity, may be a better indicator of obesity-related risk. We examined the association of overall mortality with BMI and with WC measures, including WC, waist-to-height ratio (WHtR) and waist-to-hip ratio (WHR).

**Methods:**

Data from 3976 African American participants (551 deaths) in the Jackson Heart Study (JHS) were analyzed. Cox regression models were used to perform survival analysis. Obesity measures were analyzed as dichotomous (obese/non-obese) and continuous variables. Baseline covariates included age, sex and smoking status.

**Results:**

Comparing obese to non-obese participants, adjusted hazard ratios (95% CI) for overall mortality were 1.14 (0.96, 1.35), 1.30 (1.07, 1.59), 1.02 (0.73, 1.41) and 1.45 (1.18, 1.79) when using BMI, WC, WHtR and WHR, respectively. For BMI, WC and WHtR, a J-shaped relationship was observed with overall mortality. For WHR, a monotonic increasing relationship was observed with overall mortality.

**Conclusions:**

In the JHS, we found that obesity as defined by WC and WHR was associated with an increased risk of overall and CVD mortality, while obesity defined by BMI was associated only with an increased risk of CVD mortality. WHR was the only obesity measure that showed a monotonic increasing relationship with overall and CVD mortality.

## Background

Overall mortality has been reported to be lower among individuals classified as overweight/obese based on body mass index (BMI, kg/m^2^) when compared with their normal weight counterparts (“obesity paradox”) both in the general population and in population subgroups [1–5]. In a systematic review of prospective studies of obesity and mortality among general populations of adults using normal weight (BMI 18.5 to < 25) as a reference group, overweight (BMI 25 to < 30) was associated with a significantly lower overall mortality, grade 1 obesity (BMI 30 to < 35) was not associated with a higher overall mortality, and grades 2 and 3 obesity (BMI ≥ 35) were associated with a significantly higher overall mortality [1]. Similar findings have been reported in the elderly population and among patients with chronic kidney disease, coronary heart disease, heart failure and chronic obstructive pulmonary disease [2–5].

The J-shaped relationship between BMI and overall mortality is perplexing and cannot be completely explained by potential selection biases, such as survival bias and healthy participant effect [6]. One plausible reason is that BMI does not differentiate the weight of fat mass from the weight of lean body mass or fat mass distribution phenotypes across the BMI continuum and as such is not a reliable measure of the risk of obesity-related disease. In a prospective cohort study of US male health professionals, a strong positive monotonic association was observed between predicted fat mass and overall mortality and a U-shaped association between predicted lean body mass and overall mortality, suggesting the “obesity paradox” may be largely attributable to low lean body mass, rather than low fat mass, in the lower BMI range [7]. Similarly, in a population-based cohort study of older men in UK [8], both sarcopenia and central adiposity were found to be associated with greater overall mortality, with the highest risk found in sarcopenic obese men.

Waist circumference (WC) is the most common and simplest way to measure central adiposity, which is a major contributor to disease and death. Among African Americans who are known to have relatively less of their body mass in their trunks and relatively more in their extremities compared to non-Hispanic white, WC measures of obesity may offer a more reliable assessment of obesity and mortality relationships [9]. In this study, we compared the associations of overall mortality with BMI and with other obesity measures, including WC, waist-to-height ratio (WHtR) and waist-to-hip ratio (WHR) in the Jackson Heart Study (JHS), a population-based African American cohort in the US. We hypothesized that obesity measures that incorporate waist circumference, a better measure of central adiposity and risk of obesity-related disease than BMI, will show a more consistent pattern of increasing risk of overall mortality with increasing level of obesity. In addition, we also evaluated the associations of these obesity measures with two leading causes of mortality, CVD and cancer, given the strong correlations between measures of obesity and cardiometabolic risk factors as well as risks of some cancers [10–12].

## Methods

### Data source

The design and data collection of the JHS has been previously described [13, 14]. Between September 2000 and March 2004, 5306 African Americans, ages 20 to 95 years, living in the Jackson, Mississippi metropolitan area were enrolled. Three clinical examinations were conducted between 2000 and 2013 (Exam 1 (baseline): 2000–2004; Exam 2: 2005–2008; and Exam 3: 2009–2013). Surveillance of CVD events and deaths is still ongoing.

BMI, WHtR and WHR were calculated from weight, height, waist circumference and hip circumference measurements collected during clinical exams. Weight was measured to the nearest 0.1 kg and height to the nearest centimeter in light clothing and in stocking feet; waist circumference was measured to the nearest centimeter at the umbilicus; hip circumference (HC) was measured to the nearest centimeter at the maximal protrusion. BMI was calculated as weight in kilograms divided by height in meters squared (kg/m^2^) [15]. WHtR was calculated as waist circumference divided by height. WHR was calculated as waist circumference divided by hip circumference.

Vital status of participants was ascertained through annual follow-up interviews, death records from the Mississippi State Health Department, obituaries and the National Death Index. Cause of death was assigned by applying algorithms developed by the National Center for Health Statistics (NCHS) using death certificates in accordance with the International Statistical Classification of Diseases and Related Health Problems, Tenth Revision (ICD-10) [16] and was provided by the Mississippi State Health Department. For out-of-state deaths, causes of death were based on ICD codes for the underlying cause of death recorded on death certificates or entered in the National Death Index. Causes of death were grouped according to the list of “rankable” causes of death used by the NCHS for reporting leading causes of death in the US [17].

### Analytic sample

Because hip circumference measurements were collected at Exam 2 but not at Exam 1, anthropometric data collected at Exam 2 were used for the purpose of this analysis so that the performance of all obesity measures could be compared on the same set of participants. Participants who did not return for Exam 2 (*n* = 1101), with BMI < 18.5 kg/m^2^ (*n* = 24), with missing data on any of the obesity measures (*n* = 142) or smoking status (*n* = 63) were excluded. There were 3976 participants in the analytic sample. All deaths occurring through December 31, 2016 (administrative censoring date) were included in the analysis.

### Statistical analysis

Survival functions by obesity status (obese/non-obese) were estimated using the Kaplan-Meier estimator and compared using the log-rank test. “Time 0” for the survival analyses was the date of Exam 2 and the administrative censoring date was December 31, 2016. The median length of follow-up was 9.2 (range: 0.01–11.2) years. Deaths due to CVD included cause of death with ICD-10 codes I00-I78 and deaths due to cancer included cause of death with ICD-10 codes C00-C97. Obesity status (obese/non-obese) was classified based on cut-points recommended by guidelines or reported in the literature for each obesity measure as follows: BMI ≥ 30 kg/m^2^; WC > 88 cm for women or > 102 cm for men; WHtR ≥0.5; WHR ≥ 0.85 for women or ≥ 0.9 for men [18–20].

Obesity measures were analyzed as dichotomous variables (obese/non-obese) as well as continuous variables using restricted cubic spline with 4 knots to evaluate the non-linear relationship with mortality. The knots were placed at 5th, 35th, 65th and 95th percentiles as suggested by Harrell [21]. A model with waist and hip circumferences as separate variables was also evaluated. Statistical significance of non-linearity was evaluated using log likelihood ratio test (LRT) comparing restrictive cubic spline and linear models.

Cox regression models were performed for both unadjusted analyses and analyses adjusted for age, sex and smoking status (baseline covariates). For analyses of CVD and cancer mortalities, deaths due to other causes were treated as a competing risk and were analyzed using methods proposed by Fine and Gray [22]. Age was analyzed as a continuous variable. Smoking status was classified as current/non-current smokers. Possible effect modifications by age, sex and smoking status were tested by adding three second-order interaction terms in the models. A two-sided *p*-value < 0.05 was considered statistically significant for all analyses. Sensitivity analysis excluding “early deaths,” defined as participants who died within one year of enrollment, was performed to exclude participants who may have had a low BMI due to wasting.

## Results

### Description of analytic sample

The mean age of the analytic sample (*n* = 3976) was 59.8 years, 64.5% were women and 12.4% were current smokers. The prevalence of obesity was high but varied widely depending on which measure was used to classify obesity, ranging from 55.5% (*n* = 2205) per BMI, 65.0% (*n* = 2585) per WHR, 68.4% (*n* = 2719) per WC, to 91.1% (*n* = 3622) per WHtR. The descriptive statistics of anthropometric measures of the analytic sample, overall and by subgroups, are shown in Table 1.

**Table 1:** Descriptive statistics of anthropometric measures of the analytic sample, Exam 2 (2005–2008)

	N (%)	Weight (kg) Mean (SD)	Height (cm) Mean (SD)	WC (cm) Mean (SD)	HC (cm) Mean (SD)	BMI (kg/m^**2**^) Mean (SD)	WHtR Mean (SD)	WHR Mean (SD)
**Overall**	3976 (100)	91.2 (21.1)	168.6 (9.5)	102.2 (15.7)	114.3 (14.5)	32.1 (7.1)	0.61 (0.10)	0.89 (0.08)
**Age, years**								
20–39	183 (4.6)	99.9 (29.4)	171.0 (10.0)	102.5 (20.6)	117.5 (17.6)	34.2 (9.8)	0.60 (0.12)	0.87 (0.08)
40–59	1814 (45.6)	94.4 (22.0)	169.7 (9.4)	102.1 (16.2)	115.5 (15.0)	32.8 (7.4)	0.60 (0.10)	0.88 (0.08)
≥ 60	1979 (49.8)	87.3 (18.3)	167.3 (9.3)	102.4 (14.7)	112.9 (13.5)	31.2 (6.3)	0.61 (0.09)	0.91 (0.07)
**Sex**								
Women	2564 (64.5)	88.5 (20.7)	163.6 (6.6)	101.5 (16.1)	116.6 (15.2)	33.0 (7.4)	0.62 (0.10)	0.87 (0.07)
Men	1412 (35.5)	95.9 (20.8)	177.6 (6.9)	103.6 (14.7)	110.1 (12.0)	30.3 (6.0)	0.58 (0.08)	0.94 (0.06)
**Smoking Status**								
Current smoker	493 (12.4)	88.5 (20.4)	170.6 (9.4)	100.7 (14.3)	110.4 (13.2)	30.4 (6.6)	0.59 (0.09)	0.91 (0.07)
Non-current smoker	2483 (87.6)	91.5 (21.1)	168.3 (9.5)	102.5 (15.9)	114.9 (14.6)	32.3 (7.1)	0.61 (0.10)	0.89 (0.08)
**BMI (kg/m**^**2**^**)**								
< 30	1771 (44.5)	76.1 (11.1)	169.7 (9.5)	91.1 (9.0)	103.5 (6.7)	26.3 (2.5)	0.54 (0.05)	0.88 (0.07)
≥ 30	2205 (55.5)	103.2 (19.3)	167.7 (9.3)	111.2 (14.1)	123.0 (13.2)	36.7 (6.1)	0.66 (0.08)	0.91 (0.08)

*SD* standard deviation, *BMI* body mass index, *WC* waist circumference, *HC* hip circumference, *WHtR* waist-to-height ratio, *WHR* waist-to-hip ratio

### Demographic characteristics by obesity status

For obesity per BMI, compared to non-obese participants, obese participants were younger (< 60 years: 53.8% vs. 45.7%), more likely to be women (70.8% vs. 56.6%) and less likely to be current smokers (10.5% vs. 14.8%) (Table 2). In contrast, for obesity per WC, WHtR and WHR, obese participants were older than non-obese participants. In addition, for obesity per WHR, obese participants were more likely to be men and current smokers than non-obese participants.

**Table 2:** Demographic characteristics of Jackson Heart Study Participants by obesity status, Exam 2 (2005–2008)

	BMI		WC		WHtR		WHR	
	Non-obese ***N*** = 1771 %	Obese N = 2205 %	Non-obese ***N*** = 1257 %	Obese N = 2719 %	Non-obese ***N*** = 354 %	Obese N = 3622 %	Non-obese ***N*** = 1391 %	Obese N = 2585 %
**Age, years**								
Mean (SD)	61.2 (12.6)	58.6 (11.8)	58.7 (12.8)	60.3 (11.9)	55.3 (12.8)	60.2 (12.1)	56.4 (12.2)	61.6 (11.8)
20–39	4.0	5.1	5.7	4.1	9.0	4.2	6.8	3.4
40–59	41.7	48.8	47.8	44.6	55.4	44.7	55.5	40.3
≥ 60	54.3	46.2	46.5	51.3	35.6	51.2	37.7	56.3
**Sex**								
Women	56.6	70.8	40.6	75.5	55.1	65.4	75.3	58.7
Men	43.4	29.2	59.4	24.5	44.9	34.6	24.7	41.3
**Smoking Status**								
Current smoker	14.8	10.5	14.2	11.6	17.5	11.9	9.8	13.8
Non-current smoker	85.2	89.5	85.8	88.4	82.5	88.1	90.2	86.2

*BMI* body mass index, *WC* waist circumference, *WHtR* waist-to-height ratio, *WHR* waist-to-hip ratio

### Overall mortality

#### Obesity measures as dichotomous variables (obese/non-obese)

**Fig. 1:**
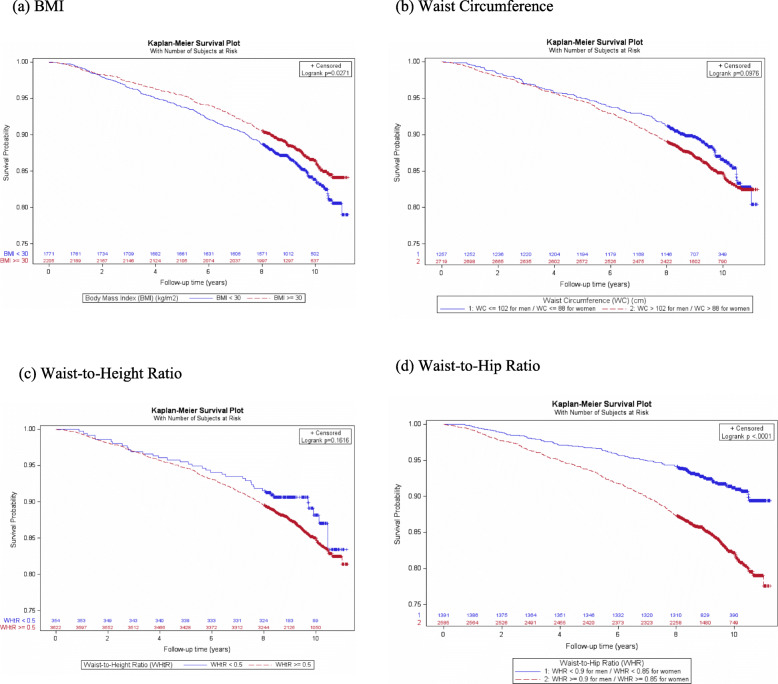
Kaplan-Meier survival plot for overall mortality by obesity status (obese/non-obese)

There were a total of 551 deaths in the analytic sample. The overall mortality rate was 15.4 per 1000 person-years. The Kaplan-Meier plots of overall mortality by obesity status (obese/non-obese) are shown in Fig. 1(a) to (d). In the unadjusted analysis, obese participants had significantly lower overall mortality compared to non-obese participants per the definition by BMI (hazard ratio (HR) 0.83 (95% CI 0.70, 0.98)) (Table 3). After adjusting for age, sex and smoking status, the adjusted HRs for overall mortality were 1.14 (95% CI 0.96, 1.35), 1.30 (95% CI 1.07, 1.59), 1.02 (95% CI 0.73, 1.41) and 1.45 (95% CI 1.18, 1.79) comparing obese to non-obese participants per definitions by BMI, WC, WHtR and WHR, respectively (Table 3, Fig. 3).

**Table 3:** Overall mortality: obese vs. non-obese

	#event/#obs	Mortality (95% CI) (/1000 Person-Years)	HR (95% CI) Unadjusted	HR (95% CI) Age-sex-smoking Adjusted
**Total**	551/3976	15.4 (14.3, 16.5)	…	…
**BMI, kg/m**^**2**^				
Non-obese	269/1771	17.0 (15.4, 18.8)	1.00	1.00
Obese	282/2205	14.1 (12.8, 15.6)	0.83 (0.70, 0.98)	1.14 (0.96, 1.35)
**WC, cm**				
Non-obese	156/1257	13.8 (12.0, 15.8)	1.00	1.00
Obese	395/2719	16.2 (14.9, 17.6)	1.17 (0.97, 1.41)	1.30 (1.07, 1.59)
**WHtR**				
Non-obese	39/354	12.3 (9.4, 16.1)	1.00	1.00
Obese	512/3622	15.7 (14.6, 16.9)	1.26 (0.91, 1.75)	1.02 (0.73, 1.41)
**WHR**				
Non-obese	114/1391	9.0 (7.7, 10.4)	1.00	1.00
Obese	437/2585	19.0 (17.5, 20.6)	2.13 (1.73, 2.61)	1.45 (1.18, 1.79)

*HR* hazard ratio, *CI* confidence interval, *BMI* body mass index, *WC* waist circumference, *HC* hip circumference, *WHtR* waist-to-height ratio, *WHR* waist-to-hip ratio

#### Obesity measures as continuous variables

**Fig. 2:**
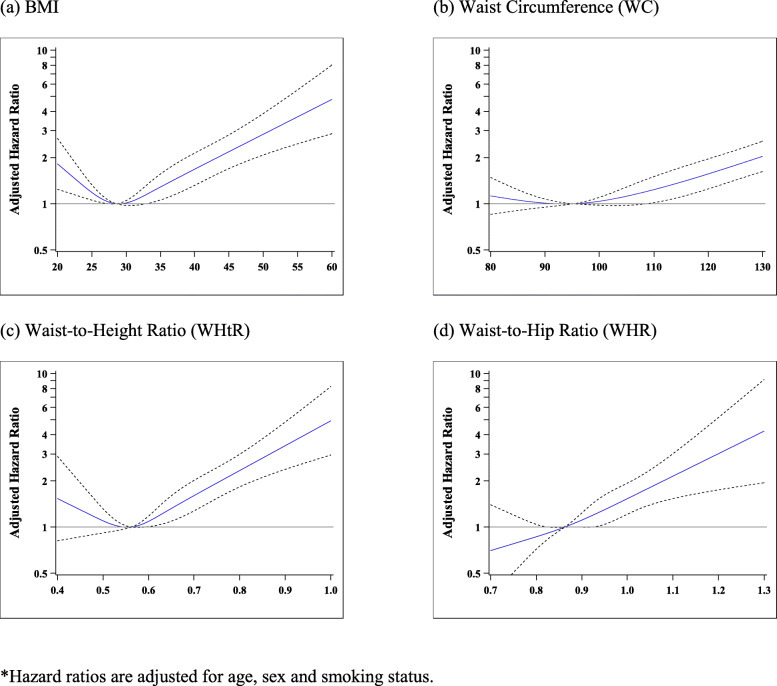
Adjusted hazard ratios* for overall mortality: analysis using restricted cubic splines

When obesity measures were analyzed as continuous variables using restrictive cubic spline, we observed, for overall mortality, a J-shaped relationship with BMI, WC and WHtR (Fig. 2(a) to (c)) and a monotonic increasing relationship with WHR (Fig. 2(d)) after adjusting for age, sex and smoking status. The improvement in model fit comparing the restricted cubic spline and linear models for WHR was not statistically significant (LRT, *p* = 0.90). In the model with waist and hip circumferences as separate variables, we observed a monotonic increase in overall mortality with increasing WC after adjusting for hip circumference and a clear decreasing trend in overall mortality with increasing hip circumference up to 120 cm after adjusting for WC. The model fit with WC (linear) and hip circumference (cubic splines) as separate variables was better than with waist-hip-ratio (linear) as a single variable (LRT, *p* < 0.001).

#### Effect modifications by age, sex and smoking status

No statistically significant interactions were found between age, sex or smoking status and obesity on mortality risk whether obesity measures were analyzed as dichotomous or continuous variables (LRT, *p* > 0.05).

#### The effect of having multiple determinants of obesity

We performed additional analysis to explore the effect of having multiple determinants of obesity on overall mortality. Only BMI, WC and WHR were included in this analysis because WHtR classified over 90% of participants as obese in our sample and was not associated with overall mortality. As shown in Table 4, the larger the number of determinants present, the greater the mortality risk. The adjusted HRs for overall mortality were 1.19 (95% CI 0.86, 1.65), 1.22 (95% CI 0.90, 1.67) and 1.54 (95% CI 1.16, 2.06) for having 1, 2 and 3 determinants, respectively. To address whether the combination of determinants matters, we further evaluated all combinations of 2 determinants of obesity and the relative mortality risks. As shown in Table 5, only the combinations that included WHR showed overall significant associations with mortality (Type 3 Test *p*-value < 0.05) (scenarios b and c). The adjusted HRs were 1.50 (95% CI 1.15, 1.97) and 1.54 (95% CI 1.17, 2.04) for combinations of BMI obese + WHR obese and WC obese + WHR obese, respectively.

**Table 4:** Overall mortality: hazard ratios by number of determinants of obesity

# Determinants Present	N (%)	HR (95% CI) Age-sex-smoking Adjusted	Type 3 Test P-value Based on Wald statistics
0	658 (16.6)	1.00 (Ref)	0.0075
1	673 (16.9)	1.19 (0.86, 1.65)	
2	1099 (27.6)	1.22 (0.90, 1.67)	
3	1546 (38.9)	1.54 (1.16, 2.06)	

*HR* hazard ratio, *CI* confidence interval

**Table 5:** Overall mortality: hazard ratios with different combinations of determinants of obesity

Scenario	# Determinants Present	N (%)	HR (95% CI) Age-sex-smoking Adjusted	Type 3 Test P-value Based on Wald statistics
**(a) BMI + WC**				
Non-obese	0	1140 (28.7)	1.00 (Ref)	0.0758
BMI obese or WC obese	1	748 (18.8)	1.12 (0.87, 1.43)	
BMI obese and WC obese	2	2088 (52.5)	1.27 (1.03, 1.57)	
**(b) BMI + WHR**				
Non-obese	0	785 (19.7)	1.00 (Ref)	0.0062
BMI obese or WHR obese	1	1592 (40.0)	1.24 (0.95, 1.62)	
BMI obese and WHR obese	2	1599 (40.2)	1.50 (1.15, 1.97)	
**(c) WC + WHR**				
Non-obese	0	722 (18.2)	1.00 (Ref)	0.0008
WC obese or WHR obese	1	1204 (30.3)	1.16 (0.86, 1.57)	
WC obese and WHR obese	2	2050 (51.6)	1.54 (1.17, 2.04)	

*HR* hazard ratio, *CI* confidence interval, *BMI* body mass index, *WC* waist circumference, *HC* hip circumference, *WHtR* waist-to-height ratio, *WHR* waist-to-hip ratio

### CVD and cancer mortalities

**Fig. 3:**
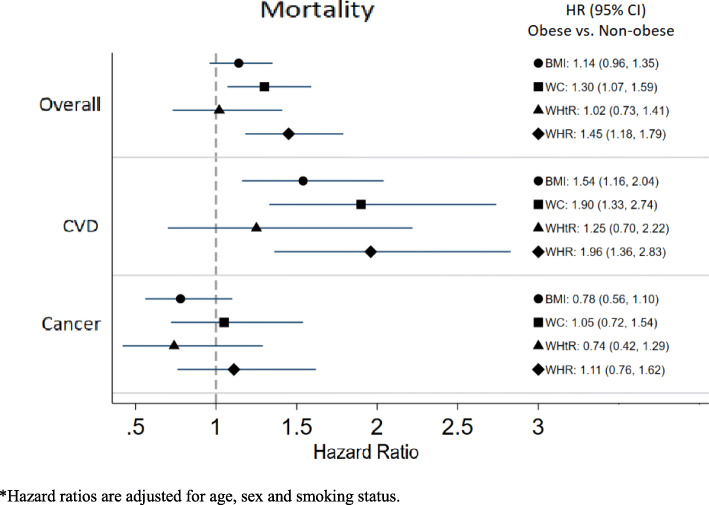
Adjusted hazard ratios* for overall, CVD and cancer mortality

There were 210 CVD deaths and 143 cancer deaths in the analytic sample. The CVD and cancer mortality rates were 5.9 and 4.0 per 1000 person-years, respectively. Obesity had a greater effect on CVD mortality than on overall mortality. The adjusted HRs for CVD mortality were 1.54 (95% CI 1.16, 2.04), 1.90 (95% CI 1.33, 2.74), 1.25 (95% CI 0.70, 2.22) and 1.96 (95% CI 1.36, 2.83) comparing obese to non-obese participants per definitions by BMI, WC, WHtR and WHR, respectively (Fig. 3). No associations were found between obesity and cancer mortality (Fig. 3).

## Discussion

Our results show that obesity classified per WC and WHR, but not per BMI or WHtR, was associated with a significantly higher overall mortality after adjusting for age, sex and smoking status. We found a J-shaped relationship between BMI, WC, WHtR and overall mortality and a linear relationship between WHR and overall mortality. We found similar results for obesity classified per BMI, WC, WHR, and WHtR and CVD mortality. However, we found no associations between any of the obesity classifications and cancer mortality.

Our findings on BMI and overall mortality were consistent with findings reported in the literature. Overweight and mild obesity were associated with a lower overall mortality compared with normal BMI (BMI < 25). This “J-shaped” association persisted even after excluding “early deaths,” defined as participants who died within one year of enrollment and thus may have low BMI due to wasting.

We found that both WHR and WC adjusted for HC, but not WC alone, were linearly associated with overall mortality. This finding suggests that not only body fat but also the distribution of body fat is important in discriminating overall mortality risk. In a recent study, using computed tomography (CT)-measured body fat, including both visceral and subcutaneous fat area, Lee et al. [23] showed that only the visceral-to-subcutaneous fat area ratio (VSR) was independently associated with overall mortality in the fully adjusted model with age, sex, comorbidities and total fat mass. In another study using data from NHANES, Dong et al. [24] demonstrated that both amount of body fat and body fat distribution (measured via WHR) were independently associated with overall mortality and the effect was sex-dependent.

Some investigators have advocated that waist and hip circumferences be considered as separate variables and not as a ratio, considering two persons with the same WHR may have markedly different levels of waist circumferences [25]. We evaluated this approach in our study and found that the model with waist and hip circumferences as separate variables outperformed the model with WHR in predicting overall mortality. These results provide further support on the interplay of the visceral and gluteofemoral fat depots and body shape on overall mortality. Possible mechanisms of the protective effect of gluteofemoral fat include long-term fatty acid storage in this location thus reducing adverse effects associated with ectopic fat deposition [26]. Gluteofemoral fat has also been found to be associated with a beneficial adipokine profile, positively associated with leptin and adiponectin levels and negatively associated with inflammatory cytokines [26].

Our analyses exploring the effect of having multiple determinants of obesity on overall mortality showed that adding BMI or WC to WHR as determinants of obesity did not substantially improve the prediction of mortality risks. Participants deemed obese by WHR were 45% more likely to die compared to those deemed not obese. Whereas participants deemed obese by both BMI and WHR were 50% more likely to die compared to those deemed obese by neither; participants deemed obese by both WC and WHR were 54% more likely to die compared to those deemed obese by neither.

Our findings of the effects of obesity on CVD mortality were similar to those on overall mortality and, as would be expected, obesity had a greater effect on CVD mortality than on overall mortality. Of note, although BMI ≥ 30 kg/m^2^ (obese) was not associated with overall mortality after adjusting for age, sex and smoking compared to BMI < 30 kg/m^2^, BMI-defined obesity was associated with a higher CVD mortality. This reaffirms the usefulness of this commonly used obesity indicator for monitoring trends of obesity and cardiovascular health.

We did not find an association between obesity and cancer mortality. It is possible that obesity-related metabolic dysregulations rather than merely obesity are required to show an association with cancer mortality [27, 28]. For example, in an analysis using data collected in the REasons for Geographic and Racial Differences in Stroke (REGARDS) study, obesity was shown to be associated with a reduced risk for cancer mortality whereas glucose dysregulation and metabolic syndrome were associated with an increased risk for cancer mortality [28].

A limitation of the study is that because JHS is a sample of African Americans, we are unable to address directly whether or not race matters in body composition or outcome in this study. It has been observed that body compositions differ across race/ethnic groups. For a given BMI, non-Hispanic (NH) blacks typically have the lowest percent fat mass (%fat) followed by NH whites and Mexican Americans have the greatest %fat [9]. NH blacks also have smaller waist circumferences than NH whites and Mexican Americans with similar BMI. Therefore, the findings of this study may not be generalizable to other race or ethnic groups.

## Conclusions

In conclusion, we found that obesity classified by WC and WHR was significantly associated with an increased risk of overall and CVD mortality in this large cohort of African Americans. Additionally, obesity classified by BMI was significantly associated with an increased risk of CVD mortality. WHR was the only obesity measure that showed a monotonic increasing relationship with overall and CVD mortality. Further studies are warranted to determine the extent to which: (1) WC and/or WHR strata can be used as mortality risk indicators in research and population health policy for African Americans as is currently being done for BMI; and (2) individuals with normal BMI but in high risk WC and/or WHR strata might represent an appreciable target population subgroup for potential lifestyle modification. Our findings suggest that WHR, a measure that captures both central adiposity and body composition, may be an important anthropometric measure to collect to monitor obesity and obesity-related risks among African Americans. These findings should be verified in other ethnically diverse populations.

## Data Availability

As a National Heart, Lung, and Blood Institute (NHLBI)-funded study, the JHS follows the NHLBI’s Policy for Data Sharing, which includes depositing the data into the NHLBI’s Biologic Specimen and Data Repository Information Coordinating Center (BioLINCC) to make it publically available to other investigators. The link to the JHS dataset: https://biolincc.nhlbi.nih.gov/studies/jhs/. Further download directions are provided on the website. NHLBI further requires the JHS to maintain the accuracy of this dataset, so the JHS Coordinating Center sends modifications to BioLINCC for incorporation as appropriate. Privacy and access settings are controlled by BioLINCC, and the investigators have no influence on these settings.

## Abbreviations

BioLINCC: Biologic Specimen and Data Repository Information Coordinating Center
BMI: Body mass index
cm: Centimeters
CI: Confidence interval
CT: Computed tomography
CVD: Cardiovascular disease
HC: Hip circumference
HR: Hazard ratio
ICD: International classification of diseases
ICD-10: International Statistical Classification of Diseases and Related Health Problems, Tenth Revision
JHS: Jackson Heart Study
kg: Kilograms
kg/m^2^: Kilograms divided by meters squared
LRT: Likelihood ratio test
NCHS: National Center for Health Statistics
NH: Non-Hispanic
NHANES: National Health and Nutrition Examination Survey
NHLBI: National Heart, Lung, and Blood Institute
NIMHD: National Institute for Minority Health and Health Disparities
REGARDS: REasons for Geographic and Racial Differences in Stroke study
SD: Standard deviation
UK: United Kingdom
US: United States
VSR: Visceral-to-subcutaneous fat area ratio
WC: Waist circumference
WHR: Waist-to-hip ratio
WHtR: Waist-to-height ratio
